# Neurocognitive assessment in obsessive compulsive disorder patients: Adherence to behavioral decision models

**DOI:** 10.1371/journal.pone.0211856

**Published:** 2019-02-15

**Authors:** Alessandra Cillo, Marco Bonetti, Giovanni Burro, Clelia Di Serio, Roberta De Filippis, Riccardo Maria Martoni

**Affiliations:** 1 Department of Decision Sciences and IGIER, Bocconi University, Milan, Italy; 2 Department of Social and Political Sciences and Dondena Research Center, Bocconi University, Milan, Italy; 3 Department of Statistics, University of Warwick, Coventry, United Kingdom; 4 University Centre of Statistics in the Biomedical Sciences, Vita-Salute San Raffaele University, Milan, Italy; 5 Department of Clinical Neurosciences, IRCCS San Raffaele Turro, Milan, Italy; Baylor University, UNITED STATES

## Abstract

In economics, models of decision-making under risk are widely investigated. Since many empirical studies have shown patterns in choice behavior that classical models fail to predict, several descriptive theories have been developed. Due to an evident phenotypic heterogeneity, obsessive compulsive disorder (OCD) patients have shown a general deficit in decision making when compared to healthy control subjects (HCs). However, the direction for impairment in decision-making in OCD patients is still unclear. Hence, bridging decision-making models widely used in the economic literature with mental health research may improve the understanding of preference relations in severe patients, and may enhance intervention designs. We investigate the behavior of OCD patients with respect to HCs by means of decision making economic models within a typical neuropsychological setting, such as the Cambridge Gambling Task. In this task subjects have to decide the amount of their initial wealth to invest in each risky decision. To account for heterogenous preferences, we have analyzed the micro-level data for a more informative analysis of the choices made by the subjects. We consider two influential models in economics: the expected value (EV), which assumes risk neutrality, and a multiple reference points model, an alternative formulation of Disappointment theory. We find evidence that (medicated) OCD patients are more consistent with EV than HCs. The former appear to be more risk neutral, namely, less sensitive to risk than HCs. They also seem to base their decisions on disappointment avoidance less than HCs.

## Introduction

Decision-making plays an essential role in daily life and comprises a complex process of assessing and weighing short-term and long-term costs and benefits of alternative actions [1]. In neurospychology, decision making has been studied with respect to both cognitive and physiological functions.

Decision-making, as a cognitive function, has been deeply investigated during the last decades as a possible marker of several psychiatric conditions. It involves an expansive network that includes the prefrontal cortex (orbitofrontal and dorsolateral prefrontal cortices), anterior cingulate cortex, thalamus, parietal cortex, and caudate [2]. The central role of dopaminergic and serotoninergic systems in decision-making is also well established [3–5]. Consistent findings are reported in several psychopathological conditions such as addictions [6], personality disorders [7], ADHD [8], eating disorders [9, 10], obsessive compulsive disorder (OCD) [11, 12] and risky behaviors [13, 14].

Focusing on OCD, in the last two decades convergent evidence from phenomenology and neurobiology has proposed OCD as a *disorder of decision-making* because of pathological doubt and a perpetuated obsessive compulsive mechanism, characterized by immediate reward with long-term punishment [15]. Decision-making contexts differ with respect to the probability distribution of the potential outcomes: under *risk* such probabilities are known, while under *ambiguity* they are not [16]. The literature provides a stable impairment in ambiguous decision-making in OCD patients [11, 12, 17–24], while results on decision-making under risk are less clear. Some authors show a deficient risky decision making in OCD patients compared to healthy control subjects (HCs), especially in choosing the most likely outcome [25]. Other authors do not find any significant difference between OCD and HC performance, suggesting that risky decision components are unimpaired in OCD patients [11, 12, 24, 26, 27].

Decisions under risk generally include comparisons between two or more risky alternatives, called *lotteries*. Consider a two-outcome lottery *L* = (*x*, *p*; *y*, 1 − *p*), that is to say, a risky alternative that can end up with an amount *x* with probability *p* or an amount *y* with probability 1 − *p*. There exist many models of decision making under risk. The expected value (EV) of the lottery *L*, namely the sum of the outcomes weighted by their probabilities of occurrence, is a well-known criterion to evaluate the lottery itself. Expected Utility (EU) theory, introduced by Bernoulli in the 1738 [28], was formalized in [29] as a way to generalize the EV criterion. Under EU, people are allowed to transform the monetary outcomes into utilities, and the evaluation of a lottery is the weighted sum of the utilities of the outcomes. The shape of the utility function reflects the risk attitude of an individual. Hence, in addition to risk neutrality (as assumed by EV), EU allows individuals to possibly be risk averse or risk prone.

Since the ’50s, many empirical studies have shown patterns in choice behavior that EU fails to predict. In a number of contexts, human decisions tend to systematically deviate from what rational choice models would predict. For example, we tend to give too much attention to irrelevant information [30], to contextual and situational variables [31], and even to rationalize our bad decisions [32]. Several behavioral models have been introduced to capture these (and more) features (see [33] for a review). Prospect theory [34, 35] is an extremely influential theory of decision under risk, and it well explains people’s attitudes towards risk [36]. One of the innovative features of Prospect theory is that individuals care about losses and gains with respect to a reference point (*status quo*). The major issue with the reference-dependent theories is how the reference point is constructed. The principal theory of reference point formation argues that the reference point is determined by people’s expectations [37, 38]. As shown in [39], the models in [38] and [40] are equivalent. In [40] a general Disappointment model with multiple reference points (MRP) is introduced. Originally, Disappointment theory assumes that people, when making decisions, minimize disappointment feelings that might arise by comparing the obtained outcome with one single reference [41, 42]. In MRP, on the other hand, people do not have a unique reference but multiple ones: the prospect itself is their reference point. This theory results from the idea that people feel both disappointed for the potential outcomes being higher than the one they received, and happy for having avoided outcomes lower than the one they received. Disappointment theory is similar to Regret theory [43], having as main difference the fact that under disappointment the comparison is made across states of nature and within the lottery, while under Regret theory the comparison is across lotteries and within states of nature. In [44] a theory of emotional experiences, Decision Affect theory (DAT), is proposed. DAT is similar to disappointment but it is not a theory of choice, rather a theory of post decision affect. It predicts that bad outcomes feel worse when unexpected than when expected, yet good outcomes feel better when unexpected than when expected. Evidence in support of DAT’s predictions is reported on in [45].

Some recent studies have investigated decision making models in patients. Patients with lesions to the orbitofrontal cortex experience attenuated regret and fail to make choices consistent with its avoidance [46]. Similarly, individuals higher on psychopathy make riskier choices and are less influenced by prospective regret when making choices [47]. Some recent evidence shows that OCD patients, despite their increased emotional responsivity to the counterfactual comparisons that characterize regret and relief, have a deficit in the use of forward counterfactual models of action-outcome, relying primarily on EV [48]. Regret and disappointment seem to share a general neural network, but they differ in both the magnitude of subjective feelings and in the intensity of activation of some regions of the brain [49]. Disappointment theory has been widely supported by experimental evidence, e.g. in [50, 51]; however, its study in psychiatric investigations is still scarse.

The goal of our work is to shed some further lights on decisions under risk for OCD patients, by combining standard models used in economics with psychiatric empirical investigation. The subjects performed the Cambridge Gambling Task, a specific task for assessing risk behavior, in which they had to decide the amount of their initial wealth to invest in a two-outcome lottery. Contrary to previous uses of the software, we extract the micro-level data for a more informative analysis of the choices made by the subjects, by accounting for heterogeneity in preferences. We focus on two major models in economics: EV, which assumes risk neutrality, and MRP, which allows for risk and disappointment aversion. For each risky decision, the two models predict different fraction of the initial wealth to be invested. We explore possible differences that may exist between OCD patients and HCs in the agreement with one of the two models, if any. We find evidence that OCD adhere to EV more than HCs. Hence, OCD patients appear to be less sensitive to risk than HCs. Disappointment comparisons guiding decision-making are less evident in OCD patients than in HCs.

## Materials and methods

131 HCs were recruited over a period of 24 months from the general population; they were recruited from the local community, administrative staff and workers of the I.R.C.C.S. San Raffaele Hospital. Axis I screening was performed with the Mini-International Neuropsychiatric Interview-Plus for DMS-IV-TR (MINI-Plus) [52] by a senior psychiatrist. 7 subjects were excluded since they met exclusion criteria, which were: presence of lifetime brain injuries (N = 1), neurological diseases, and/or presence of an actual/lifetime psychiatric diagnosis (Lifetime Major Depressive Episode = 3; Binge Eating Disorder = 1; Panic Disorder = 2). The final sample contained 124 HCs.

For what concerns the OCD patients, as per exclusion criterion, no patients with psychotic spectrum diseases, current/past severe brain injuries, or IQ below standard, were allowed to participate in the study. 85 OCD patients were recruited over the same period as HCs from consecutive admissions to the Department of Clinical Neurosciences at I.R.C.C.S. San Raffaele Turro in Milan. Diagnosis of OCD was made by senior psychiatrists according to APA guidelines [53]. Four patients were excluded because they dropped out of the evaluation of symptoms severity. Treated patients received selective serotonin reuptake inhibitors (SSRIs) or SSRIs plus low-dose antipsychotics and/or mood stabilizers and/or clomipramine. Symptoms and severity of illness were measured using the Dimensional Yale-Brown Obsessive-Compulsive Scale (DY-BOCS) [54]. Age of onset and duration of illness were also collected. Comorbidities were reported for 6 patients. One patient had a history of social phobia, four of mood disorders, and one of dysmorphophobia. The final sample contained a total of 81 OCD patients.

Note that the sample size of this study (81 OCD patients and 124 healthy controls) was not designed to target a pre-specified difference between the two groups. The fact that we were able to identify statistically significant effects through the models that we have implemented suggests that the study was indeed adequately powered.

All subjects gave their written informed consent after a detailed explanation of the procedure. This study was conducted in accordance with the Code of Ethics of the World Medical Association (Declaration of Helsinki) for experiments involving humans, and the Milan Area Health Authority Ethics Committee approved the study.

The data can be accessed by contacting the Authors. Statistical analyses were performed using Statistica 8.0 [55], STATA 13 [56], and R 3.5.1 [57].

### The task

The 81 OCD patients and 124 HCs were assessed with the Cambridge Gambling Task (CGT), a specific computer-based task designed to assess decision-making and risk-taking behaviour outside a learning context. The CGT is part of the standard battery of the Cambridge Neuropsychological Test Automated Battery (CANTAB) software [58].

**Fig 1:**
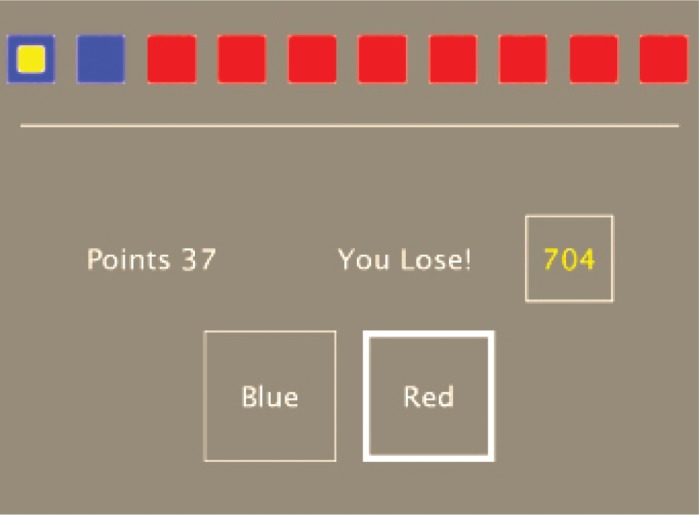
Screenshot from the Cambridge Gambling Task. Screenshot from the CGT. In this example, the subject bet on red, but the blue won. He/She lost 37 points ending up with 704 points.

The task included different stages. At the beginning of each stage, instructions were provided. Each stage was composed by several bets. At each bet, the subject was presented with a row of ten boxes, some of which red and some of which blue. By touching the appropriate box at the bottom of the screen, the subject guessed whether a winning yellow token was hidden in a red or in a blue box. The subject started with 100 points (initial endowment) displayed on the screen. For each bet he/she was asked to select a fraction of the current endowment to bet: 5%, 25%, 50%, 75% or 95%. After the fraction was selected, one of the ten boxes became yellow, so that the winning color was revealed. Hence, if the subject had bet on the color of that box, he/she gained the amount bet, with the final endowment being the endowment of the previous stage plus the gained points. Instead, if the yellow box did not appear on the color that the subject had chosen, then he/she lost the amount bet, with the final endowment being the endowment of the previous stage minus the lost points. Then the subject moved to the next bet, with the initial endowment of the successive bet equal to the final endowment of the previous one. Fig 1 shows an example of the task.

The fractions that the subject could bet were presented alternatively in two different ways: from lowest to highest percentage (ascending condition) or from highest to lowest percentage (descending condition). All subjects bet under both conditions throughout the assessment, and the order in which the two conditions were presented (ascending first or descending first) was balanced.

Each subject had to face a total of seven stages. The first two were simulation stages, each of which consisted of four bets. Then, there were two assessed stages, up to nine bets per stage, in which subjects were presented in an increasing order the possible fractions of their endowment to bet. Next, subjects were presented in a decreasing order the possible fractions of the endowment to bet, both for one simulation stage consisting of four bets and for two assessed stages. Hence, the assessed bets were at most 36 (9 bets per each of the four assessed stages) per subject. The experiment ended sooner for 20% of HCs and 36% of OCD patients. These subjects ended up, by rounding, with zero points, because they had bet a high fraction of a small endowment.

The decisions were hypothetical in nature. Indeed, subjects were not paid a flat fee for the task, neither played for real one of the choices. Despite some evidence that monetary incentives might or might not provide the appropriate incentives (see [59] and [60]), in case of gains, playing out for real at least one choice might improve the quality of data. When losses are involved, things become more complicated. If one were to play for real, she would need to provide the subjects with an initial endowment, which they could eventually lose. However, the procedure of potential losses from an initial endowment has been criticized from a theoretical viewpoint [61]. In addition, in the loss domain the behavior of subjects as a function of the payment scheme has been tested, and no significant difference between hypothetical and real conditions was found [62].

### Two models in decision making

Under EU, the shape of the utility function reflects the risk attitude of an individual. A concave (convex) function represents a risk averse individual (risk prone). A linear utility function would coincide exactly with EV, namely, the individual would be classified as a risk neutral person.

The expected evaluation of a lottery *L* might incorporate possible disappointment (or elation) feelings that might arise when comparing the outcome obtained with all other possible higher (or lower) outcomes of the lottery [40]. Let *x*_1_ ≥ *x*_2_ ≥ …. ≥ *x*_*n*_ be the outcomes of a lottery, and *p*_*i*_ the probability of receiving outcome *x*_*i*_, *i* = 1, …*n*. The expected *evaluation* of the lottery *L* is defined as:

$$\begin{array}{c} U\left(L\right)=\sum_{i=1}^{n}p_{i}v\left(x_{i}\right)-\sum_{i=1}^{n}\sum_{j\geq i}p_{i}p_{j}H\left(v\left(x_{i}\right)-v\left(x_{j}\right)\right), \end{array}$$ (1)

where *v*(*x*_*i*_) represents the evaluation of outcome *x*_*i*_, and *H*(.) quantifies how individuals value discrepancies between obtained and missed outcomes (see Eq (12) in [40] and page 765 in [63]). Precisely, *H*(.) captures the difference between the disappointment and the elation that each outcome is able to trigger when compared to better and worse outcomes, respectively. We refer to the model in Eq (1) as the multiple reference points (MRP) model. Under the EV criterion, one sets *v*(*x*) = *x*. Under MRP we assume *v*(*x*) = 1 − *exp*(−0.017*x*) and *H*(*y*) = *y* + *e*^−*y*^ − 1 (as in [64] and on page 770 in [63]). The specific form of the *H*(.) function satisfies the theoretical requirements as first and second order stochastic dominance, and second order risk aversion [63]. Under these assumptions, both EV and MRP would suggest betting on the color with the highest probability. EV would always recommend that one bets 95% of the initial points on the color with the highest probability, independently on what this probability is. The EV criterion assumes that the decision maker is insensitive to risk. On the other hand, the MRP model *modulates* the amounts to bet. It assumes that people might prefer more conservative strategies in order to avoid possible disappointment feelings that might arise in case of a loss. As the probability of winning increases, the MRP model suggests to invest a higher proportion of points, up to 75%, on the color with the highest probability: the predicted fractions to bet clearly depend also on the current endowment. There are few circumstances (5% of the choices) in which also the MRP predicts to bet 95%: for simplicity, these bets were excluded from the analysis.

## Results

The CGT produces the following default outcome measures: (i) Quality of Decision Making (QDM), the measure of the proportion of bets in which the subject chose to gamble on the color with the highest probability; (ii) Deliberation Time, the mean latency from presentation of the colored boxes to the subject’s choice of which color to bet on; (iii) Risk Taking (RT), the mean proportion of the total points (between 5% and 95%) that the subject chose to bet when he/she had chosen the color with the highest probability; (iv) Overall Proportion Bet, the average proportion of the current total points (between 5% and 95%) that the subject chose to risk on each bet, including when betting on the color with equal or lowest probability; (v) Risk Adjustment, which measures the tendency to bet a higher proportion of points when the large majority of the boxes are of the chosen color than when a smaller majority of the boxes are of the chosen color.

Table 1 describes the basic demographic features of 81 OCD patients and 124 HCs in the sample.

**Table 1:** Demographic variables in OCD patients and HCs. Means (standard deviations in parentheses).

	OCD	HCs	Sig. (*p* < 0.05)
N	81	124	
Age (years)	33.5 (11.8)	32.1 (11.1)	Not Sig.
Education (years)	14.1 (2.9)	14.8 (3)	Not Sig.
Percent Female	0.395 (0.054)	0.556 (0.045)	Sig.

Pairwise unadjusted Least Significant Differences were produced and tested to assess the effects of sex and diagnosis on the outcome variables, as shown in Table 2. The last column in Table 2 summarizes the significant differences that emerged from this preliminary analysis, which was performed on the summary measures produced routinely by the CANTAB software. Only the quality of decision-making did not significantly differ between OCD patients and HCs, while for all the other measures there was a difference between the two groups. Specifically, OCD patients were betting significantly more than HCs regardless of probability (Overall Proportion Bet), and also when betting on the color with the highest probability (Risk Taking). Importantly, the Risk Adjustment measure was significantly higher for HCs than OCD patients. Moreover, the deliberation time was different between the two groups: OCD patients revealed longer deliberation time than HCs. We can hypothesize that this result might be due to the clinical characteristics of pathological doubt and perpetuated obsessive-compulsive that lengthens the time of a decision mechanism [15]. Lastly, differences by sex were only observed for the Risk Taking measure, and only for the ascending choices.

**Table 2:** Neuropsychological variables in OCD patients and HCs by sex (M,F). Means (standard deviations in parentheses). (1): OCD-M vs. HC-F; (2): OCD-M vs. HC-M; (3): OCD-F vs. HC-M; (4): all OCD vs. all HC (note: the p-values *p* are unadjusted).

	OCD-M	OCD-F	HC-M	HC-F	OCD	HC	*p* < 0.05
N	49	32	55	69	81	124	
Quality of Decision Making (DM)	0.83 (0.17)	0.84 (0.15)	0.85 (0.16)	0.88 (0.13)	0.83 (0.16)	0.87 (0.14)	
Quality of DM (Asc.)	0.78 (0.20)	0.83 (0.16)	0.80 (0.20)	0.85 (0.14)	0.80 (0.19)	0.83 (0.17)	
Quality of DM (Desc.)	0.87 (0.16)	0.85 (0.20)	0.90 (0.13)	0.91 (0.16)	0.87 (0.18)	0.91 (0.15)	
Risk taking	**0.54 (0.15)**	0.47 (0.14)	**0.47 (0.15)**	**0.44 (0.14)**	**0.51 (0.15)**	**0.45 (0.15)**	(1)(2)(4)
Risk taking (Asc.)	**0.43 (0.20)**	**0.35 (0.19)**	**0.39 (0.15)**	**0.35 (0.15)**	0.40 (0.20)	0.37 (0.15)	(1)(3)
Risk taking (Desc.)	0.64 (0.22)	0.65 (0.23)	0.54 (0.21)	0.52 (0.19)	**0.64 (0.22)**	**0.53 (0.20)**	(4)
Deliberation time	3304 (1697)	3461 (2846)	2866 (1123)	2726 (900)	**3366 (2208)**	**2788 (1003)**	(4)
Deliberation time (Asc.)	3847 (2488)	3791 (2776)	3167 (1301)	3048 (1110)	**3825 (2589)**	**3101 (1195)**	(4)
Deliberation time (Desc.)	2728 (1150)	3102 (3164)	2549 (1121)	2397 (976)	2876 (2169)	2464 (1042)	
Overall proportion bet	0.48 (0.14)	0.45 (0.13)	0.42 (0.15)	0.41 (0.14)	**0.47 (0.14)**	**0.41 (0.14)**	(4)
Overall proportion bet (Asc.)	0.39 (0.18)	0.33 (0.17)	0.35 (0.15)	0.33 (0.14)	0.37 (0.18)	0.34 (0.14)	
Overall proportion bet (Desc.)	0.60 (0.22)	0.61 (0.23)	0.51 (0.21)	0.49 (0.19)	**0.60 (0.22)**	**0.50 (0.19)**	(4)
Risk adjustment	0.97 (1.38)	0.48 (1.61)	1.23 (1.06)	1.09 (1.05)	**0.78 (1.48)**	**1.15 (1.05)**	(4)
Risk adjustment (Asc.)	0.89 (1.56)	0.56 (1.26)	1.19 (1.34)	1.05 (1.36)	0.76 (1.45)	1.11 (1.35)	
Risk adjustment (Desc.)	0.90 (1.58)	0.71 (1.08)	1.31 (1.26)	1.19 (1.18)	**0.82 (1.40)**	**1.24 (1.21)**	(4)

To be able to detect differences between OCD patients and HCs in terms of adherence to one of the two decision-making models, we extracted the individual subject data for each bet. In particular, the choices made at each assessed bet by each subject were compared to the optimal amount predicted by EV and MRP.

We compared agreement with the optimal bets predicted by the EV model or the MRP model, with respect to the (baseline) choice of any other bet. Specifically, we defined agreement as the bet being identical to the optimal bet that was predicted by the EV or MRP model, among the five possibilities that were allowed (5%, 25%, 50%, 75% or 95% of the total amount owned). The ten boxes can be either red or blue. Let *Boxes* be the number of boxes, among the 10 boxes shown on the screen, with the highest probability color. For example, 6 means that there were 6 boxes of the same color, and 4 of the other one. Therefore, the chance of winning when betting on the highest probability color is 60%. Both theories predict that subjects should bet on the most probable color. We excluded all cases with *Boxes = 5*, and initially performed four separate analyses of the bets performed when presented with *Boxes* = *6, 7, 8*, or *9*. We used multinomial regression models to explore the relationships between the observed agreement with the models and the covariates Sex (Male vs. Female), Order (Ascending vs. Descending), and the two indicator variables HC (124 subjects) and CaseNoMed (non medicated OCD patients, 10 subjects) vs. the baseline group of the medicated OCD patients (70 subjects). One subject was removed from these analyses since information on medication was missing. We decided not to include the duration of the illness, available for 74 patients, since it was self reported by the patients and therefore, due to the nature of OCD, expected to be very unreliable.

A complete model selection procedure was performed, including or removing direct effects and pairwise interaction terms for the two logit components of the multinomial regression model [65]. Specifically, for each case a stepwise AIC-based model selection process was performed starting from the model with all main effects and all pairwise interaction terms. To avoid overfitting, such model was then refined by performing repeated approximate likelihood ratio tests to remove any unnecessary terms (we used an exclusion threshold of 0.1). We also manually removed the Male x CaseNoMed interaction term given that it was based on very small numbers of subjects. All final models were then fitted with robust standard error estimation.

The results of the model selection procedure and model fitting are reported in Table 3, together with the estimated parameters. Some preliminary considerations emerge. Table 3 suggests the following direct effects of covariates on the agreement with the EV model: (i) Males adhere more than females to EV for *Boxes = 7*, 8, and 9; (ii) Adherence to EV is lower for ascending presentation than for descending presentation across all values of *Boxes*. Indeed, EV predicts to always bet 95%, independently on the values of Boxes. In a descending presentation, 95% is the first fraction that subjects see, in the ascending it is the last one. Hence, betting 95% in an ascending presentation requires a longer waiting time, and this might be the reason why the adherence to EV is lower for the ascending presentation; (iii) Medicated OCD patients adhere more than controls to EV across all *Boxes*. Similarly, in terms of agreement with the MRP model we note that: (i) Males and females do not adhere differently to MRP; (ii) Adherence to MRP is higher for ascending presentation than for descending presentation across all values of *Boxes*, except for *Boxes = 9*. This is coherent with the fact that MRP most of the times suggests to bet no more than 50% of the current endowment; (iii) Medicated and non medicated OCDs do not appear to behave differently in terms of their agreement with the MRP model; (iv) Overall, the comparison of medicated OCD patients’ adherence to MRP when compared to HC is not conclusive. The only significant effect (for *Boxes = 7*) suggests that HCs follow MRP more than (medicated) OCD patients. We choose not to try and interpret the emerging interaction terms.

**Table 3:** Stratum-specific multinomial regression models for the probabilities of agreement with EV and MRP. Estimated parameters (std errors). HC is equal to one for healthy controls and CaseNoMed is equal to one for non medicated OCD patients, so that the reference group is that of medicated OCD patients. Baseline outcome is no agreement with either model. *:p<0.1; ^△^:p<0.05; ^†^:p<0.01.

	Boxes = 6		Boxes = 7		Boxes = 8		Boxes = 9	
	EV	MRP	EV	MRP	EV	MRP	EV	MRP
Male	0.320 (0.30)	0.209 (0.21)	0.689^△^ (0.29)	-0.119 (0.19)	0.375* (0.22)	-0.170 (0.18)	0.447* (0.24)	0.075 (0.18)
Ascending	-1.882^†^ (0.34)	0.444^†^ (0.15)	-1.427^†^ (0.26)	0.471^†^ (0.15)	-1.720^†^ (0.22)	0.507^†^ (0.16)	-1.857^†^ (0.32)	-0.643* (0.35)
CaseNoMed	-0.406 (0.80)	-0.270 (0.49)			-1.167^△^ (0.58)	-0.883 (0.54)	-0.545 (0.46)	-0.989 (0.82)
HC	-0.960^†^ (0.31)	-0.175 (0.22)	-0.806^†^ (0.28)	0.348* (0.20)	-0.749^†^ (0.23)	0.263 (0.22)	-0.533* (0.28)	-0.234 (0.31)
Asc × CaseNoMed					2.138^†^ (0.78)	1.457^△^ (0.69)	0.569 (0.63)	1.604* (0.96)
Asc × HC							-0.344 (0.42)	0.733* (0.41)
Constant	-1.503^†^ (0.30)	-1.760^†^ (0.23)	-1.661^†^ (0.28)	-1.906^†^ (0.21)	-0.631^†^ (0.23)	-1.367^†^ (0.25)	0.011 (0.26)	-0.721^†^ (0.25)

The consistent directions of the effects observed in the stratified analyses suggest that conducting a pooled analysis of all data in a larger model that includes *Boxes* as a covariate may be reasonable. Table 4 shows the results of the global model after the model selection process. Such model allows one to confirm and expand on the conclusions obtained from the stratified analyses. The global model confirms the main effects of Ascending, HC/CaseNoMed, and Male. Indeed, (i) Males adhere more than females to EV; (ii) Adherence to EV (MRP) is lower (higher) for ascending than descending presentation; (iii) Medicated OCD patients adhere more to EV than HCs and non medicated OCD patients. This analysis expands on previous results by adding the direct effect of *Boxes*: (iv) The higher *Boxes*, the higher the probability of adherence to EV and MRP. These effects are modified by the five interaction terms *Male×CaseNoMed*, *Ascending×CaseNoMed*, *Ascending×HC*, *Boxes×Ascending*, and *HC×Boxes*.

**Table 4:** Multinomial regression model for the probabilities of agreement with EV and MRP. Estimated parameters (standard errors in parentheses). Baseline outcome is no agreement with either model. *Boxes* is the number of boxes, among the 10 boxes shown on the screen, with the highest probability color; HC is equal to one for healthy controls and CaseNoMed is equal to one for non medicated OCD patients, so that the reference group is that of medicated OCD patients. *:p<0.1; ^△^:p<0.05; ^†^:p<0.01.

	EV	MRP
Boxes	0.553^†^ (0.08)	0.232^△^ (0.10)
Male	0.379* (0.19)	-0.007 (0.12)
Ascending	-1.299 (0.84)	1.123^△^ (0.57)
HC	-1.997^△^ (0.96)	-0.849 (0.77)
CaseNoMed	-2.594^†^ (0.94)	-0.501 (0.38)
Male×CaseNoMed	2.179^△^ (0.99)	-0.090 (0.37)
Ascending×CaseNoMed	1.015* (0.54)	0.931* (0.48)
Ascending×HC	-0.304 (0.33)	0.345* (0.21)
Boxes×Ascending	-0.049 (0.10)	-0.138* (0.08)
HC×Boxes	0.165 (0.12)	0.106 (0.10)
Constant	-5.039^†^ (0.70)	-3.098^†^ (0.75)

**Fig 2:**
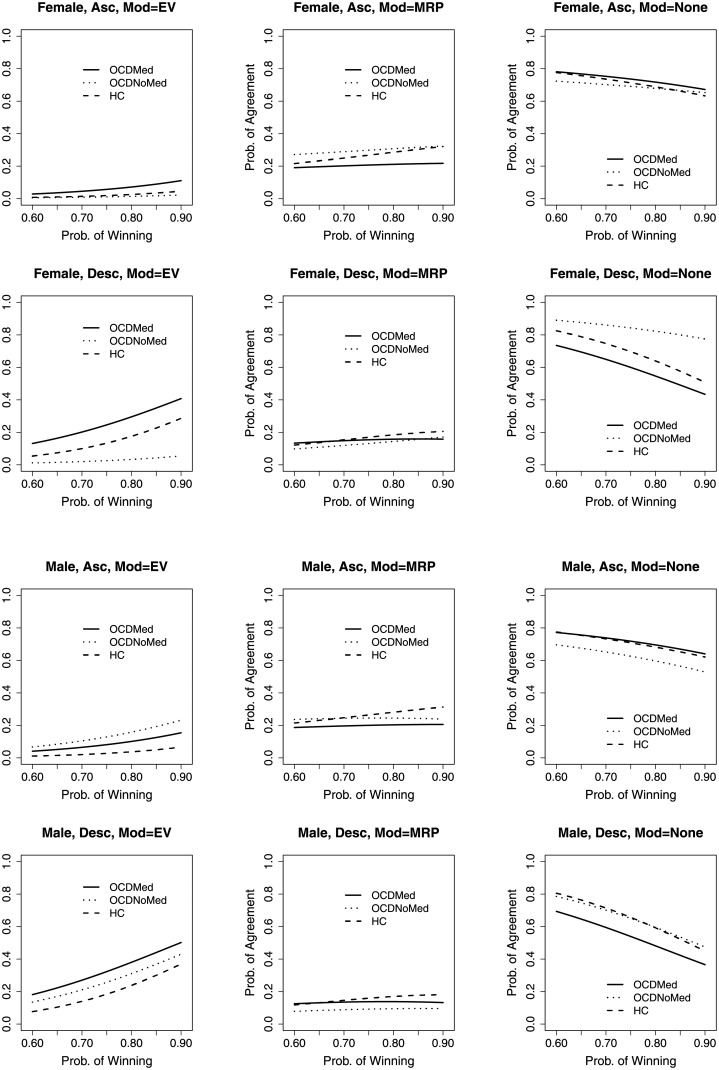
Estimated probabilities of agreement to models. *Prob. of Winnning* indicates the probability of winning as measured by the frequency of the chosen color. *Prob. of Agreement* indicates the probability of agreement to a model (EV, MRP, None). Each figure has *Prob. of Agreement* as function of *Prob. of Winnning* for medicated OCD patients, non medicated OCD patients, and for HCs.

Note, however, that the interpretation of parameters in multinomial regression models (and their significance) is global across all values for the outcome variable. Indeed, the absolute effect of covariates on the probability of agreeing with the two models (vs. neither one) requires the explicit calculation of such probabilities as a function of the covariates from the estimated parameter values. Hence, we compute for all combinations of sex and order of presentation, the model predicted probabilities of adherence to the two decision models (or to neither). We show these predictions, separately for medicated OCD patients, non medicated OCD patients, and HCs, in Fig 2. As the probability of winning increases, the probability of agreement to EV is always increasing, and it increases more in the descending than in the ascending condition. Medicated OCD patients always agree more than HCs to EV. In the descending condition, the difference in terms of probability of agreement to EV between medicated OCD patients and HCs is relevant (difference of almost 20%) and constant as the probability of winning increases, while in the ascending condition such difference is small. In terms of agreement to MRP, HCs tend to adhere more than medicated OCD patients. Medicated OCD patients’ probability of agreement to MRP is almost constant and around 20%. Also, medicated OCD patients are less influenced by higher probabilities than HCs, namely, probability seems to matter less for them. In particular, for HCs in the ascending condition, the probability of agreement to MRP slightly increases in the probability of winning, ranging from a minimum of about 20% to a maximum of about 30% (an agreement higher than what was found in [39]).

Overall, the evidence about the group of non medicated OCD patients is mixed, but the small size of that group suggests caution in interpreting those results.

To assess goodness of fit of the model, in Table 5 we report the observed frequencies of bets (choices) that agreed with EV, MRP, or neither model, within the three groups of HCs, medicated OCD patients, and non medicated OCD patients. A comparison of Table 5 and Fig 2 confirms an excellent fit of the model to the observed proportions of agreement, for all three treatment groups.

**Table 5:** Numbers and percentages of bets in agreement with EV, MRP, or none for HCs, medicated OCD patients (Med), and non medicated OCD patients (NoMed), across the four groups defined by the combination of sex and ascending vs. descending presentation of the bets.

	N. of Bets			EV			MRP			None		
	HC	Med	NoMed	HC	Med	NoMed	HC	Med	NoMed	HC	Med	NoMed
Female-Ascending	1048	420	46	0.02	0.05	0.00	0.28	0.21	0.33	0.70	0.74	0.67
Female-Descending	1002	342	48	0.15	0.26	0.04	0.16	0.13	0.10	0.69	0.60	0.85
Male-Ascending	836	604	118	0.03	0.10	0.14	0.25	0.19	0.23	0.72	0.71	0.63
Male-Descending	799	531	120	0.20	0.32	0.27	0.16	0.14	0.10	0.64	0.54	0.63

Lastly, we have examined the probability of betting on the lower probability color. Note that this is the same event summarized by the Quality of Decision Making measure provided by the CANTAB software (see Table 2). None of the variables are significant in a logistic regression model that describes the probability of making that choice, except for *Boxes*: higher values of *Boxes* were associated with a lower probability of betting on the low probability color, with a decrease of about 10% from *Boxes = 6* to *Boxes = 9*.

In the S1 Appendix we report on some additional robustness analyses.

## Discussion

The CANTAB software has been widely used in neuropsychological experiments to decision making. However, it suffers from the limitation that results are provided at an aggregate level. Indeed, the summary outcome measures produced routinely by the software only allow for partial analyses, possibly stratified by ascending vs. descending presentation and by groups of subjects (see, e.g. Table 2). Here, we have extracted and analyzed the micro-level data that CANTAB uses to produce those outcome measures. Indeed, the agreement with the optimal amount to bet according to a decision model can only be studied by looking at each choice of each subject. In addition, the individual data allow for a more informative analysis of the choices made by each subject, considering heterogeneity in preferences, while taking into account several variables at once, interaction terms and, importantly, the probability of winning as measured by the frequency of the chosen color. Formal model selection procedures can be implemented, and analyses can be performed while taking into account the dependence that exists among decisions made by the same subject, thus providing more robust inference and conclusions.

Our main results have to do with medicated OCD patients, and below for simplicity we refer to them just as OCD patients. Such patients adhere significantly more than HCs to EV. This result suggests two considerations. First, the fact that OCD patients tend to bet the highest and same proportion of points more often than HCs, can be interpreted as evidence in favor of impulsive and compulsive (habitual) behavior, respectively. Similarly, evidence exists that OCD patients have a deficit in goal-directed control and an over-reliance on habits [66]. Second, OCD patients show a lower sensitivity to risk than HCs. From an experimental point of view, extremely high levels of risk aversion are in general observed, while prescriptive decision models suggest that people should be less risk averse and more risk neutral.

OCD patients adhere less to MRP than HCs. While it is out of the scope of the current paper to test how sensitive OCD patients are to disappointment feelings, we can state that OCD patients seem to be less guided than HCs by models that minimize disappointment emotions (as MRP). This result is in line with what has been observed in the regret literature for OCD patients [48, 66] and for patients with an orbitofrontal cortex lesion [46].

A very well documented phenomenon in behavioral economics is that people tend to overweight small probabilities and underweight large ones. Several theories, from [35] to [67], can account for such phenomenon. These probability transformations can well describe subjects’ risk aversion [68]. For OCD patients, the probability of agreement to MRP as a function of the probability of winning (number of boxes) is constant and equal to approximately 20%. Hence, OCD patients are less influenced by such probability than HCs. This result might therefore be interpreted as a reduced sensitivity to risk.

The gold standard cognitive behavioural therapy (CBT) proposal for psychological intervention with OCD patients is the technique of Exposure with Response Prevention (ERP), which is associated with large improvement in symptoms [69]. In ERP, patients are asked to expose to their fear (the obsessive thought and/or the environmental cues that trigger it) without engaging in the compulsive behavior, which is habitually performed to reduce anxiety. Traditionally, the efficacy of ERP is explained by habituation that takes place when a patient is exposed to the anxiety-provoking cue for an extended period of time: the extinction learning takes place when the person sees that the imagined feared consequence does not occur. In [70] a cognitive perspective is proposed, which is not necessarily in contrast with the traditional one. Dual learning systems theory suggests that refraining from a compulsive act (an existing habit) requires using the parallel model-based/goal-directed system; the perspective developed by [71] suggests that this switch necessarily depends on the arbitration system. In particular, they provide evidence for the existence in the human brain of an arbitrator mechanism that determines the extent to which model-based (goal-directed) and model-free (habitual) learning systems control behavior. Specifically, this arbitrator proportionately allocates behavioral control through the degree of reliability of the two systems. Different studies underlined that the balance between goal-directed and habitual systems might sometimes break down in diseases such as OCD [48, 66]. Based on these perspectives, our results could be in agreement with the evidence that cognitive dysfunctions in OCD seem to be present both in situations in which learning is required to establish a new behavioral pattern, and when patients are directed to follow a new instruction. Indeed, our patients showed more adherence to the EV model, highlighting difficulties in adapting their decision to the probability. For this reason, given the existing theory and our results, the exercise of this arbitration system (in addition to extinction) could indeed be an important mechanism of change in the ERP process.

Our findings suggest some topics for further research. In this task EV predicts to bet always the same amount. It would be interesting to explore adherence to EV in another task in which the prediction of the model varies. This would help in disentangling, within OCD patients, between their habitual behavior component and their reduced sensitivity to risk. Second, the observed difference between ascending and descending presentation might suggest that the order has an impact on decision making. Developing a task in which subjects directly insert the amount they are willing to bet might reduce this effect in the data. Third, note that we have extracted the subject-specific information recorded during the CANTAB administration of the test from the backup files created by the same program. As a consequence, our approach can be replicated on similar series collected on other populations of interest and extended to other relevant questions, such as the study of differential learning processes. Finally, this study might be extended to future work that combines decision making with functional magnetic resonance imaging (fMRI), in order to deepen the neural mechanism involved during and before the decision making process in OCD patients and in HCs.

## Supporting information

S1 Appendix Additional analyses. (PDF)
