# Supplementary material for: Neurocognitive assessment in obsessive compulsive disorder patients: Adherence to behavioral decision models
Source: PLoS One. 2019 Feb 15;14(2):e0211856. doi: 10.1371/journal.pone.0211856 (PMC6377126; doi:10.1371/journal.pone.0211856)
Supplement: S1 Appendix — (PDF) [file pone.0211856.s001.pdf]

Supporting Information

Neurocognitive Assessment in Obsessive Compulsive Disorder Patients: Adherence to Behavioral Decision Models

Alessandra Cillo[1], Marco Bonetti[2,*], Giovanni Burro[3], Clelia Di Serio[4], Roberta De Filippis[5], Riccardo Maria Martoni[5]

1 Department of Decision Sciences and IGIER, Bocconi University, Milan, Italy
2 Department of Social and Political Sciences and Dondena Research Center, Bocconi University, Milan, Italy
3 Department of Statistics, University of Warwick, Coventry, UK
4 University Centre of Statistics in the Biomedical Sciences, Vita-Salute San Raffaele University, Milan, Italy
5 Department of Clinical Neurosciences, IRCCS San Raffaele Turro, Milan, Italy
* marco.bonetti@unibocconi.it

Some additional analyses

This document contains the results of some additional analyses, either preliminary or prompted by the Reviewers. We first report on some preliminary analyses that did not distinguish between medicated and non medicated OCDs. Similarly to what appears in the main text, S1 Table A shows the parameter estimates of the model, and S1 Fig A

S1 Table A. Multinomial regression model for the probabilities of agreement with EV and MRP. Estimated parameters (standard errors in parentheses). Baseline outcome is no agreement with either model. Case is equal to one for OCD patients (regardless of medication status), so that HC is the reference group. *Boxes* is the number of boxes, among the 10 boxes shown on the screen, with the highest probability color. *:p<0.1; $^{\triangle}$:p<0.05; †:p<0.01.

	EV	MRP
Male	0.43$^{\triangle}$ (0.21)	0.15 (0.17)
Ascending	-1.83$^{\triangle}$ (0.84)	1.64† (0.59)
Case	2.14$^{\triangle}$ (0.96)	0.88 (0.77)
Boxes	0.72† (0.09)	0.34† (0.08)
Male×Ascending	0.18 (0.32)	-0.25 (0.19)
Case×Ascending	0.41 (0.31)	-0.16 (0.20)
Boxes×Ascending	-0.03 (0.10)	-0.15* (0.08)
Boxes×Case	-0.20* (0.12)	-0.13 (0.10)
Constant	-7.05† (0.73)	-4.06† (0.62)

shows the estimated probabilities of agreement for the two groups of subjects (HCs vs. Cases). The results show that grouping all cases together produces estimates of agreement that fall close to those for the group of medicated cases in the main text.

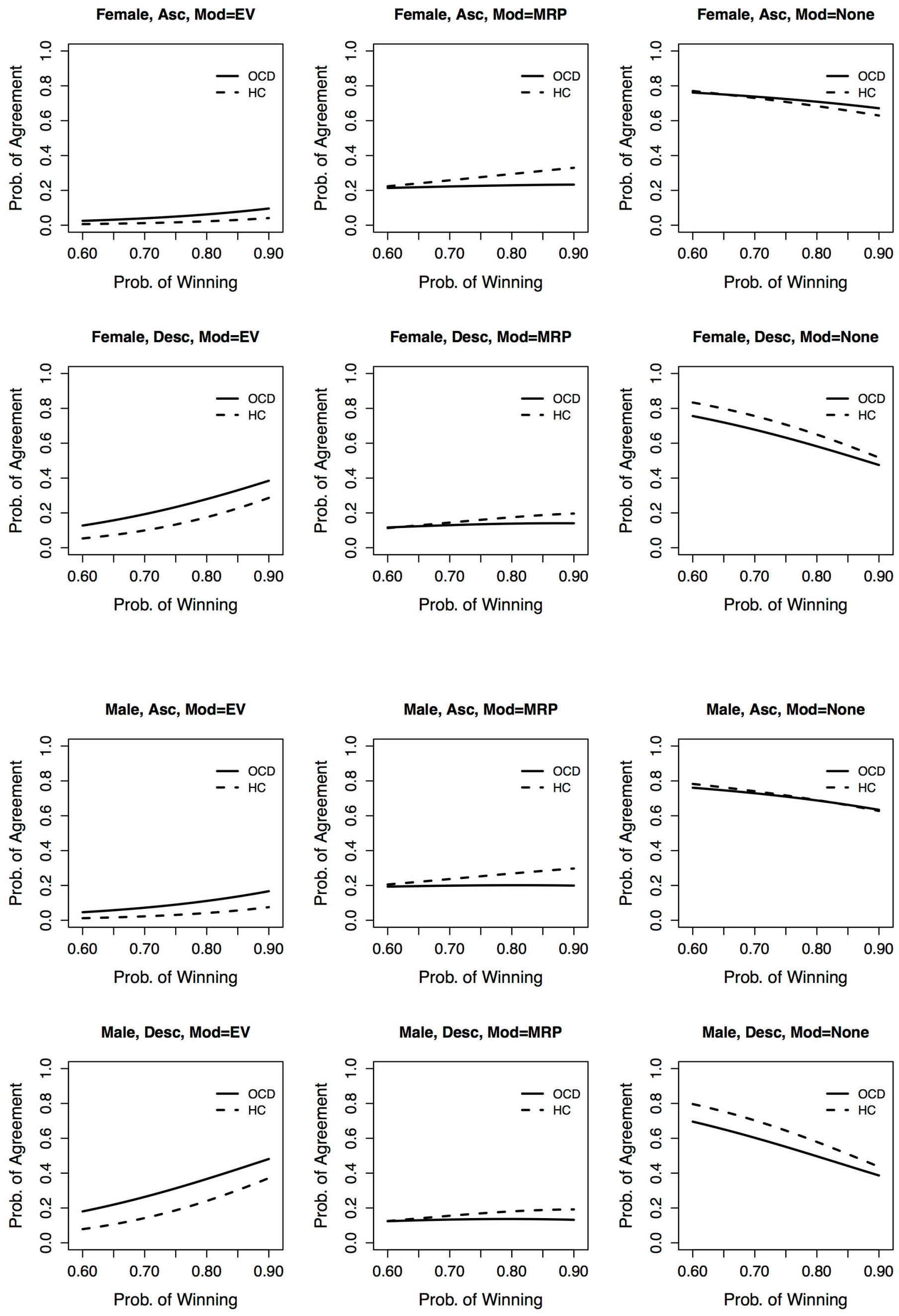

S1 Fig A. Estimated probabilities of agreement to models. *Prob. of Winnning* indicates the probability of winning as measured by the frequency of the chosen color. *Prob. of Agreement* indicates the probability of agreement to a model (EV, MRP, None). Each figure shows the *Prob. of Agreement* as function of *Prob. of Winnning*, for OCD patients (regardless of medication status) and for HCs.

S1 Table B. Multinomial regression model for the probabilities of agreement with EV *only*, MRP *only*, MV *only*, or with more than one model among these. Estimated parameters (standard errors in parentheses). Baseline outcome is no agreement with any of the models. HC is equal to one for healthy controls and CaseNoMed is equal to one for non medicated OCD patients, so that the reference group is that of medicated OCD patients. Case is equal to one for OCD patients, so that the reference group is that of HCs. *Boxes* is the number of boxes, among the 10 boxes shown on the screen, with the highest probability color. *:p<0.1; $^{\triangle}$:p<0.05; †:p<0.01.

	EV	MRP	MV	More than one
Boxes	0.0409 (0.10)	0.0636 (0.08)	-0.567† (0.08)	0.560† (0.08)
Male	0.411* (0.24)	-0.0336 (0.12)	-0.139 (0.15)	0.516† (0.18)
Ascending	-2.351† (0.31)	0.292† (0.10)	-0.744† (0.12)	-1.672† (0.16)
CaseNoMed	-0.534 (0.53)	-0.262 (0.34)	0.390 (0.27)	-0.719* (0.42)
HC	-1.101 (1.16)	-0.518 (0.74)	1.647$^{\triangle}$ (0.75)	-2.075$^{\triangle}$ (0.88)
HC×Boxes	0.0354 (0.15)	0.0989 (0.10)	-0.232$^{\triangle}$ (0.11)	0.184* (0.11)
CaseNoMed×Ascending	1.440$^{\triangle}$ (0.63)	0.613 (0.43)	-0.259 (0.50)	0.930 (0.57)
Constant	-1.962$^{\triangle}$ (0.77)	-1.943† (0.59)	2.463† (0.55)	-5.390† (0.62)

As shown in Table 5 of the article, the majority of the subjects' choices agree with neither model. While here we have compared EV to MRP, other models that account for the many remaining choices may drive subject behavior even more. In this document we present the results of an analysis that also includes a third model, the well known Mean-Variance (MV) model, which emerges from assuming a different specification of the $H(\cdot)$ function [1, Eq.13]. The value used for the β parameter in the MV model is one widely used in the literature, 0.0011, (see [2]). Moreover, it agrees with first order stochastic dominance (FSD) in 3/4 of the choices, to be coherent with a choice of H for the MRP model, which fully agrees with FSD and the utility function.

We report on the results from such analyses in S1 Table B and in S1 Fig B. Note that S1 Fig B refers to female subjects (results for male subjects are very similar). Note that considering several models does not allow us to properly disentangle the effects of the models. Specifically, the definition of the outcome variables needs to be changed, so that in S1 Table B the first three outcomes are defined as the bet matching the optimal bet from a given model *only*. Indeed, the best discriminating power is achieved when one considers models with little or no overlap in their predicted bets. Comparing a large number of competing models would require an experimental setting in which the models' predictions do not overlap too much, so that subjects' differential agreement with each model can be disentangled.

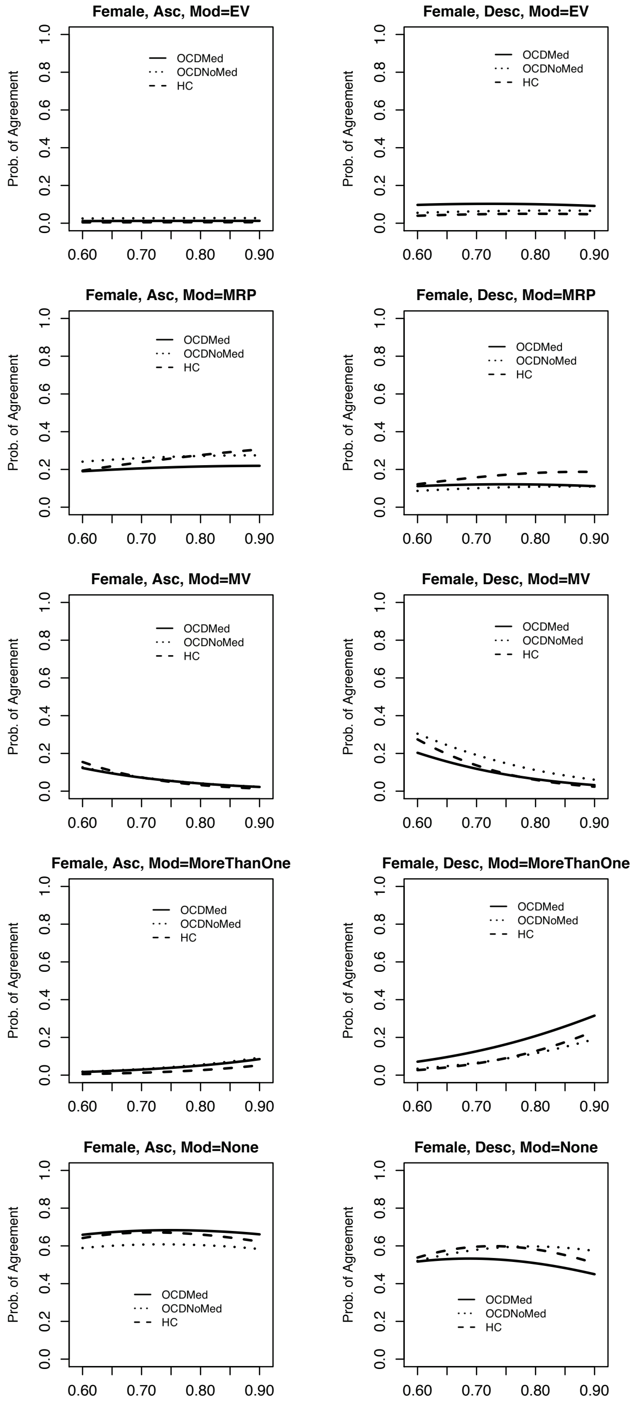

S1 Fig B. Estimated probabilities of agreement to models. *Prob. of Winnning* indicates the probability of winning as measured by the frequency of the chosen color. *Prob. of Agreement* indicates the probability of agreement to a model (EV *only*, MRP *only*, MV *only*, or with more than one model among these). Each figure shows the *Prob. of Agreement* as function of *Prob. of Winnning*, both for OCD patients (-) and HC (- -).

Lastly, we explore the sensitivity of our results to the value of the parameter in the utility function in the MRP model. So far, we have assumed, as suggested by the literature, $v(x) = 1 - exp(-\lambda x)$, with $\lambda = 0.017$.

The magnitude of λ reflects risk aversion: the higher its value, the higher the risk aversion. As λ changes, the optimal choices suggested by MRP can change. Moreover, the set of choices we take into account changes, since we exclude those cases where MRP optimal choice is betting 95% of the points (since that would overlap with EV and we would not be able to disentangle between the two models). In our sensitivity analysis, we re-estimated Table 4 by varying the value of the parameter λ. First, as S1 Table C(I) shows, we make the parameter λ (common to all subjects) assume four additional different values, namely, 0.011, 0.014, 0.020 and 0.023. Second, as S1 Table C(II) shows, we assume $\lambda_1 = 0.017$ for HCs and non medicated OCDs, while we let λ_2 vary for the medicated OCDs (0.011, 0.014, 0.020, and 0.023). Note that we have assigned the same parameter value to the two groups that in our work were found to behave similarly. Lastly, we keep $\lambda_2 = 0.017$ for medicated OCDs, and allow λ_1 to vary for the other group (HCs and non medicated OCDs) (see S1 Table C(III)).
Note that in the first four cases the value of the λ parameter is the same for OCD patients and for HCs. In the remaining cases, we have allowed λ to take different values between the two groups of subjects. That means that some of the difference in behaviour between the two groups may now be due to the different λ, and that may have the effect of changing the value of the regression parameter corresponding to the group indicator in the multinomial models. As such, these latter analyses should be approached with some caution, and indeed we have included them here only for completeness.
As we can see in S1 Table C, our conclusions remain basically unchanged as far as consistency with EV is concerned. All the coefficients have the same effect and significance only barely changes.

As far as concerns the consistency with MRP, we note that the interpretation of the coefficient for HC is robust to different model specifications. Consistency of the non medicated OCD patients with MRP remains relatively stable, with significant changes when we assume a lower level of risk aversion for non medicated OCD patients and HCs. In that case, non medicated OCDs are consistently more adherent to MRP. However, we recall that the inference on non medicated OCDs is based on a small set of choices. When we assume a higher level of risk aversion for HCs and non medicated OCDs, we can no longer claim that the ascending presentation has an impact on non medicated OCD patients. Finally, when we assume a lower risk aversion for all subjects (S1 Table C(I)) we note that the impact of the interaction effect between HCs and ascending presentation decreases and the impact of the interaction effect between HCs and number of boxes increases.

S1 Table C. Multinomial regression model for the probabilities of agreement with EV and MRP. Estimated parameters (standard errors in parentheses). Baseline outcome is no agreement with either model. *Boxes* is the number of boxes, among the 10 boxes shown on the screen, with the highest probability color; HC is equal to one for healthy controls and CaseNoMed is equal to one for non medicated OCD patients, so that the reference group is that of medicated OCD patients. We present four models, which vary in the value of λ. The first value (λ_1) refers to the parameter value chosen for HCs and non medicated OCD patients, while the second value (λ_2) is the parameter value chosen for medicated OCD patients. *:p<0.1; $^{\triangle}$:p<0.05; †:p<0.01.

(I)	$\lambda_1=\lambda_2=0.011$		$\lambda_1=\lambda_2=0.014$		$\lambda_1=\lambda_2=0.020$		$\lambda_1=\lambda_2=0.023$	
	EV	MRP	EV	MRP	EV	MRP	EV	MRP
Boxes	0.633† (0.09)	0.320† (0.09)	0.608† (0.08)	0.353† (0.01)	0.534† (0.08)	0.177* (0.10)	0.514† (0.08)	0.129 (0.10)
Male	0.359* (0.19)	0.024 (0.11)	0.366* (0.19)	0.026 (0.11)	0.379* (0.19)	0.025 (0.13)	0.390$^{\triangle}$ (0.20)	0.005 (0.13)
Ascending	-0.819 (0.89)	2.090†(0.49)	-1.036 (0.86)	2.209† (0.50)	-1.393* (0.84)	0.834 (0.59)	-1.529* (0.85)	0.686 (0.65)
HC	-1.746† (1.00)	-0.274 (0.68)	-2.055$^{\triangle}$ (0.98)	-0.455 (0.73)	-1.993$^{\triangle}$ (0.94)	-1.059 (0.78)	-2.030$^{\triangle}$ (0.95)	-1.255 (0.78)
CaseNoMed	-2.441† (0.89)	0.259 (0.29)	-2.401† (0.93)	0.405 (0.29)	-2.586† (0.94)	-0.434 (0.43)	-2.579† (0.92)	-0.536 (0.45)
Male×CaseNoMed	2.138$^{\triangle}$ (0.93)	-0.248 (0.38)	2.003$^{\triangle}$ (0.97)	-0.648$^{\triangle}$ (0.32)	2.167$^{\triangle}$ (0.99)	-0.242 (0.40)	2.143$^{\triangle}$ (0.97)	-0.313 (0.41)
Asc×CaseNoMed	0.866* (0.56)	0.071 (0.31)	0.937* (0.55)	0.210 (0.31)	1.024* (0.54)	1.038$^{\triangle}$ (0.51)	1.057* (0.56)	1.299$^{\triangle}$ (0.59)
Ascending×HC	-0.555 (0.34)	0.021 (0.18)	-0.391 (0.34)	0.160 (0.17)	-0.318 (0.33)	0.398* (0.21)	-0.305 (0.33)	0.399* (0.21)
Boxes×Ascending	-0.108 (0.10)	-0.262† (0.07)	-0.086 (0.01)	-0.283† (0.07)	-0.038 (0.10)	-0.105 (0.08)	-0.021 (0.10)	-0.085 (0.08)
HC×Boxes	0.144 (0.12)	0.072 (0.09)	0.180 (0.12)	0.086 (0.09)	0.164 (0.12)	0.122 (0.10)	0.169 (0.12)	0.151 (0.10)
Constant	-5.603† (0.72)	-3.573† (0.68)	-5.418† (0.70)	-3.915† (0.72)	-4.901† (0.69)	-2.751† (0.75)	-4.775† (0.69)	-2.465† (0.78)
(II)	$(\lambda_1,\lambda_2)=(0.017,0.011)$		$(\lambda_1,\lambda_2)=(0.017,0.014)$		$(\lambda_1,\lambda_2)=(0.017,0.020)$		$(\lambda_1,\lambda_2)=(0.017,0.023)$	
	EV	MRP	EV	MRP	EV	MRP	EV	MRP
Boxes	0.597† (0.09)	0.205$^{\triangle}$ (0.09)	0.581† (0.08)	0.259† (0.10)	0.543† (0.08)	0.190$^{\triangle}$ (0.10)	0.530† (0.08)	0.130 (0.10)
Male	0.354* (0.20)	0.011 (0.12)	0.358* (0.20)	0.001 (0.12)	0.381* (0.20)	0.006 (0.13)	0.383* (0.20)	0.014 (0.12)
Ascending	-1.269 (0.88)	0.756 (0.55)	-1.390 (0.85)	1.068* (0.56)	-1.328 (0.84)	0.996* (0.58)	-1.346 (0.84)	0.903 (0.58)
HC	-1.748* (0.99)	-1.066 (0.71)	-1.980$^{\triangle}$ (0.97)	-0.764 (0.74)	-2.041$^{\triangle}$ (0.95)	-1.004 (0.77)	-2.107$^{\triangle}$ (0.95)	-1.294* (0.77)
CaseNoMed	-2.653† (0.93)	-0.721* (0.38)	-2.639† (0.93)	-0.604 (0.38)	-2.573† (0.94)	-0.411 (0.39)	-2.551† (0.93)	-0.306 (0.39)
Male×CaseNoMed	2.191$^{\triangle}$ (0.98)	-0.138 (0.37)	2.168$^{\triangle}$ (0.98)	-0.102 (0.37)	2.175$^{\triangle}$ (0.99)	-0.106 (0.37)	2.172$^{\triangle}$ (0.99)	-0.118 (0.37)
Asc×CaseNoMed	1.038* (0.56)	0.978$^{\triangle}$ (0.46)	1.090$^{\triangle}$ (0.55)	0.967$^{\triangle}$ (0.47)	1.014* (0.54)	0.933* (0.48)	1.008* (0.54)	0.915* (0.48)
Ascending×HC	-0.475 (0.34)	0.318 (0.2)	-0.339 (0.34)	0.358$^{\triangle}$ (0.18)	-0.302 (0.33)	0.350 (0.22)	-0.305 (0.33)	0.336 (0.21)
Boxes×Ascending	-0.047 (0.10)	-0.082 (0.08)	-0.038 (0.10)	-0.130* (0.08)	-0.045 (0.10)	-0.122 (0.08)	-0.042 (0.10)	-0.108 (0.08)
HC×Boxes	0.128 (0.12)	0.110 (0.10)	0.159 (0.12)	0.082 (0.10)	0.173 (0.12)	0.138 (0.10)	0.185 (0.12)	0.190* (0.10)
Constant	-5.339† (0.72)	-2.715† (0.68)	-5.220† (0.70)	-3.204† (0.73)	-4.980† (0.70)	-2.873† (0.75)	-4.903† (0.70)	-2.520† (0.75)
(III)	$(\lambda_1,\lambda_2)=(0.011,0.017)$		$(\lambda_1,\lambda_2)=(0.014,0.017)$		$(\lambda_1,\lambda_2)=(0.020,0.017)$		$(\lambda_1,\lambda_2)=(0.023,0.017)$	
	EV	MRP	EV	MRP	EV	MRP	EV	MRP
Boxes	0.623† (0.08)	0.322† (0.10)	0.594† (0.08)	0.314† (0.10)	0.549† (0.08)	0.212$^{\triangle}$ (0.10)	0.548† (0.08)	0.214$^{\triangle}$ (0.10)
Male	0.352* (0.19)	0.002 (0.12)	0.364* (0.19)	0.019 (0.12)	0.381* (0.19)	0.002 (0.13)	0.375* (0.20)	-0.026 (0.13)
Ascending	-0.821 (0.88)	2.279† (0.51)	-1.062 (0.86)	2.200† (0.52)	-1.341 (0.84)	0.944 (0.59)	-1.373 (0.84)	0.826 (0.63)
HC	-1.788* (0.99)	-0.106 (0.75)	-2.123$^{\triangle}$ (0.97)	-0.598 (0.77)	-1.952$^{\triangle}$ (0.95)	-0.886 (0.78)	-1.920$^{\triangle}$ (0.95)	-0.822 (0.80)
CaseNoMed	-2.383† (0.89)	0.494* (0.29)	-2.373$^{\triangle}$ (0.93)	0.531* (0.30)	-2.582† (0.95)	-0.476 (0.44)	-2.603† (0.93)	-0.673 (0.45)
Male×CaseNoMed	2.141$^{\triangle}$ (0.93)	-0.227 (0.38)	2.006$^{\triangle}$ (0.97)	-0.642$^{\triangle}$ (0.32)	2.152$^{\triangle}$ (1.00)	-0.239 (0.420)	2.146$^{\triangle}$ (0.98)	-0.301 (0.42)
Asc×CaseNoMed	0.861 (0.56)	0.045 (0.31)	0.929* (0.55)	0.177 (0.32)	1.018* (0.54)	1.006* (0.52)	1.049* (0.56)	1.280$^{\triangle}$ (0.60)
Ascending×HC	-0.564* (0.34)	-0.004 (0.20)	-0.400 (0.34)	0.135 (0.20)	-0.310 (0.33)	0.357* (0.21)	-0.311 (0.33)	0.373* (0.21)
Boxes×Ascending	-0.107 (0.10)	-0.284† (0.07)	-0.081 (0.10)	-0.279† (0.07)	-0.043 (0.10)	-0.114 (0.08)	-0.039 (0.10)	-0.098 (0.08)
HC×Boxes	0.157 (0.12)	0.082 (0.12)	0.193 (0.09)	0.122 (0.10)	0.155 (0.12)	0.092 (0.10)	0.149 (0.11)	0.074 (0.10)
Constant	-5.578† (0.71)	-3.826† (0.75)	-5.340† (0.69)	-3.748† (0.75)	-5.009† (0.70)	-2.959† (0.75)	-5.002† (0.70)	-2.955† (0.78)

References

1. Delquié P, Cillo A (2006). Disappointment without prior expectation: A unifying perspective on decision under risk. *Journal of Risk and Uncertainty*, 33, 197-215.

2. Sargent TJ, Ljunggvist L (2004). Recursive Macroeconomic Theory. The MIT Press.
